# Integration of the Draft Sequence and Physical Map as a Framework for Genomic Research in Soybean (*Glycine max* (L.) Merr.) and Wild Soybean (*Glycine soja* Sieb. and Zucc.)

**DOI:** 10.1534/g3.111.001834

**Published:** 2012-03-01

**Authors:** Jungmin Ha, Brian Abernathy, William Nelson, David Grant, Xiaolei Wu, Henry T. Nguyen, Gary Stacey, Yeisoo Yu, Rod A. Wing, Randy C. Shoemaker, Scott A. Jackson

**Affiliations:** * Interdisciplinary Life Science Program, Purdue University, West Lafayette, Indiana 47907; † Institute of Plant Breeding, Genetics & Genomics, Center for Applied Genetic Technologies, University of Georgia, Athens, Georgia 30602; ‡ BIO5 Institute, University of Arizona, Tucson, Arizona 85721; § United States Department of Agriculture–Agricultural Research Service Corn Insects and Crop Genetics Research Unit, Ames, Iowa 50011; ** National Center for Soybean Biotechnology, Division of Plant Sciences, University of Missouri, Columbia, Missouri 65211

**Keywords:** FingerPrinted Contig, whole-genome sequencing, genome structure, genome evolution

## Abstract

Soybean is a model for the legume research community because of its importance as a crop, densely populated genetic maps, and the availability of a genome sequence. Even though a whole-genome shotgun sequence and bacterial artificial chromosome (BAC) libraries are available, a high-resolution, chromosome-based physical map linked to the sequence assemblies is still needed for whole-genome alignments and to facilitate map-based gene cloning. Three independent *G. max* BAC libraries combined with genetic and gene-based markers were used to construct a minimum tiling path (MTP) of BAC clones. A total of 107,214 clones were assembled into 1355 FPC (FingerPrinted Contigs) contigs, incorporating 4628 markers and aligned to the *G. max* reference genome sequence using BAC end-sequence information. Four different MTPs were made for *G. max* that covered from 92.6% to 95.0% of the soybean draft genome sequence (gmax1.01). Because our purpose was to pick the most reliable and complete MTP, and not the MTP with the minimal number of clones, the FPC map and draft sequence were integrated and clones with unpaired BES were added to build a high-quality physical map with the fewest gaps possible (http://soybase.org). A physical map was also constructed for the undomesticated ancestor (*G. soja*) of soybean to explore genome variation between *G. max* and *G. soja*. 66,028 *G. soja* clones were assembled into 1053 FPC contigs covering approximately 547 Mbp of the *G. max* genome sequence. These physical maps for *G. max* and its undomesticated ancestor, *G. soja*, will serve as a framework for ordering sequence fragments, comparative genomics, cloning genes, and evolutionary analyses of legume genomes.

With recent advances in sequencing technology, whole-genome sequencing projects are becoming routine. Several years ago, the legume research community recommended soybean as model genome for Phaseoloid legumes (Gepts *et al.* 2005) because of its agronomical importance and existing genomic infrastructure. Shortly thereafter, physical mapping and whole-genome shotgun sequencing efforts for soybean were undertaken resulting in a genome sequence for soybean (*Glycine max*) (Schmutz *et al.* 2010) followed by the resequencing of its undomesticated ancestor, *Glycine soja* Sieb. and Zucc. (Kim *et al.* 2010). Even with a genome sequence, a physical map may still be needed to correctly locate DNA sequences to specific chromosomes, especially because the current short-read sequencing technologies are problematic in obtaining reliable ordering of complete chromosome assemblies as the result of repetitive sequences, large gene families, and segmental duplications that cannot be spanned by the short sequence reads (Lewin *et al.* 2009).

Clone-based maps have been integral to several genome sequencing projects, contributing to gene cloning, the understanding of genome structure, and evolutionary studies. McPherson *et al.* illustrated the benefit of using the clone-based physical map in the assembly of the human genome sequence (McPherson *et al.* 2001). A physical map also contributed to the sequencing of the *Drosophila melanogaster* genome (Hoskins 2000), and a combination strategy of physical mapping and sequencing was applied to the mouse genome (Bouck *et al.* 2000; Pennisi 2000). To support the increasing interest in map-based gene cloning of important genes, the physical map of *Arabidopsis thaliana* was constructed, resulting in deeper understanding of genome structure and evolution (Mozo *et al.* 1999). Rice genome sequencing data were integrated with a physical map, and this integrated high-resolution physical map facilitated genome sequencing through a minimal tiling path of BAC clones (Chen *et al.* 2002). To build a foundation to sequence the maize genome, physical and genetic maps of maize were developed and anchored to each other, resulting in an useful tool for evolutionary studies of maize (Cone *et al.* 2002; Wei *et al.* 2007; Wei *et al.* 2009).

For soybean, physical maps were constructed using BAC libraries from cv. Forrest and cv. Faribault (Wu *et al.* 2004a,b). However, the community selected the cultivar Williams82 for a reference genome sequence. A high-quality physical map was needed as a foundation to improve the usefulness of the whole genome sequence for the research community. An initial physical map for Williams 82 was derived from two BAC libraries made with different restriction enzymes (Pampanwar *et al.* 2005; Soderlund *et al.* 2000; Warren 2006). This map consisted of 97,272 fingerprinted BAC clones comprising 1893 contigs and approximately 30,000 singletons. The physical map needed to be integrated with the genome sequence and oriented with the genetic map to identify genes underlying quantitative trait loci, which is important for the genetic improvement of soybean and to understand the molecular and genetic basis of traits (Jackson *et al.* 2006). To improve the genetic anchoring of physical map of *G. max*, 3290 microsatellites (simple sequence repeat [SSR]) markers were identified from BAC end sequences (BES) of clones comprising the initial physical map and 265 of these SSR were genetically mapped (Shoemaker *et al.* 2008).

The genomes of *G. max* and *G. soja* have been sequenced using whole-genome shotgun sequencing, *G. max* with traditional Sanger sequencing, and *G. soja* with next-generation sequencing. In both instances, a physical map can be used to improve the genome sequence by spanning gaps and correcting alignments. Wild soybean, *G. soja*, is a promising source of genes/alleles that were lost during domestication bottlenecks (Hyten *et al.* 2006). Thus, the physical map of *G. soja* will be useful to clone potentially valuable genes, to improve the quality of the *G. soja* genome sequence, and as a foundation for comparative evolutionary studies.

For the *G. max* physical map, a minimum tiling path (MTP) can be picked using BESs aligned to the genome sequence. Traditionally, the main purpose of a MTP has been to efficiently select clones to be sequenced; in other words, to minimize the number of clones to be sequenced by selecting clones that are adjacent and overlap minimally. In the case of *G. max*, in which the whole-genome shotgun data are available, the primary purpose of the MTP is to have a physical map anchored to the genome sequence, thereby providing a framework for genomic research. A reliable MTP covering nearly the whole genome complements a genome shotgun sequence in that it can be used to correct misalignments and to span gaps, which is important for finishing regions and cloning genes. For *G. soja*, the physical map provides an anchored, clone-based resource to shuttle between the two genomes, domesticated and undomesticated.

## Materials and Methods

### Source BAC libraries

The DNA source for soybean BAC libraries was from the cultivar Williams 82 that has been chosen as the standard genotype by the soybean community for genomic studies (Stacey *et al.* 2004). Three different restriction enzymes *Hind*III, *Bst*yI, and *Eco*RI, were used to construct the three libraries, GM_WBa, GM_WBb, and GM_WBc, respectively (Table 1). The DNA for *G. soja* BAC library, GSS_Ba, was from a single plant of accession PI468916, and *Hind*III was used to construct the library (Table 1).

**Table 1:** Summary of soybean BAC libraries used in the FPC maps

Species	Library	Restriction Enzyme	Avg. Insert Size, kb	Genome Equivalents Coverage	No. of Clones	No. of Clones Fingerprinted
	GM_WBa	*Hin*dIII	150	5.4x	40,320	35,145
*G. max*	GM_WBb	*Bst*yI	150	12.0x	91,160	61,379
	GM_WBc	*Eco*RI	131	10.9x	92,160	37,658
*G. soja*	GSS_Ba	*Hin*dIII	150	12.5x	92,160	81,247

BAC, bacterial artificial chromosome; FPC, FingerPrinted Contigs.

### Source of sequences

Assembly of shotgun sequenced fragments in soybeans presents substantial challenges because of the duplicated nature of the genome (Shoemaker *et al.* 1996), many repeat sequences, and common domains of several gene families. Although the shotgun sequencing data (gmax 1.01) has several fold coverage of the entire genome, 377 gaps remain (Schmutz *et al.* 2010). We integrated 950,068,807 bp of sequence length from the 20 pseudomolecules with the physical map (Table 2).

**Table 2:** Sequence coverage length of four different MTPs of *G. max*

Scaffold	Gmax1.01	Gaps (1000 N Arachne Scaffolds)	FPC Clones/Paired BES	FPC Clones/Unpaired BES	All Clones/Paired BES	All Clones/Unpaired BES
Gm01	55,915,595	14	54,031,028	54,244,841	54,433,357	54,601,601
Gm02	51,656,713	26	46,688,786	47,183,562	47,929,653	48,513,213
Gm03	47,781,076	26	43,827,475	44,370,246	44,853,580	45,265,110
Gm04	49,243,852	15	46,627,725	46,846,968	47,116,298	47,312,649
Gm05	41,936,504	10	40,348,170	40,564,085	40,845,469	41,053,468
Gm06	50,722,821	27	46,260,437	46,788,486	47,211,351	47,644,800
Gm07	44,683,157	14	41,102,917	41,164,938	41,920,695	42,048,769
Gm08	46,995,532	12	43,259,780	43,501,037	43,820,537	44,082,436
Gm09	46,843,750	14	44,028,454	44,385,184	44,620,246	44,965,599
Gm10	50,969,635	30	46,425,807	46,591,044	47,456,533	47,653,723
Gm11	39,172,790	20	36,518,365	36,952,892	37,127,519	37,458,495
Gm12	40,113,140	21	36,686,674	37,102,118	37,428,667	37,907,123
Gm13	44,408,971	24	38,222,478	38,577,480	38,771,342	39,016,163
Gm14	49,711,204	13	46,563,799	46,777,020	47,097,234	47,295,050
Gm15	50,939,160	20	47,896,452	48,328,091	48,564,594	48,828,076
Gm16	37,397,385	23	33,365,708	33,564,017	34,143,930	34,511,921
Gm17	41,906,774	15	38,264,930	38,544,807	39,073,164	39,268,910
Gm18	62,308,140	25	58,408,218	58,891,175	59,710,660	60,015,568
Gm19	50,589,441	17	47,831,756	48,094,335	48,742,366	48,956,251
Gm20	46,773,167	11	43,107,226	43,451,533	43,160,043	43,575,687
Total	950,068,807	377	879,466,185	885,923,859	894,027,238	899,974,612
Additional coverage from unpaired BES				6,457,674		5,947,374

MTP, minimum tiling path; FPC, FingerPrinted Contigs; . BES, BAC end sequences.

### Source of MTP

Because the gmax 1.01 soybean assembly did not filter out clones with unusually long or short inserts, we limited BAC lengths to a range of 75 kb to 225 kb when MTPs were picked from two different clone pools; one pool contained only BAC clones, which were used to construct the FingerPrinted Contigs (FPC) map (clone pool A), and the other contained all the BAC clones from the three BAC libraries (clone pool B). Two kinds of MTPs were picked from each clone pool by using Dijkstra’s shortest path algorithm (Dijkstra 1983). One MTP was picked from only the BAC clones with paired BESs and the other from BAC clones with both paired and unpaired BESs in order to try and extend coverage into sequence gaps (Figure 2).

### Spanning gaps in the FPC map

To span the gaps in the preliminary FPC map having 1893 contigs, the map was integrated with a preliminary 4x sequence assembly from the Joint Genome Institute and the Stanford Human Genome Center. The average length of contigs was 157,040 bp, and the maximum size was 20,109,437 bp (Batzoglou *et al.* 2002; Jaffe *et al.* 2003). The integration was performed using the BSS and MTP modules of FPC as described in Nelson and Soderlund (2009). The 148 spanned gaps (contig merges) were automatically identified and performed by FPC (Table 3).

**Table 3:** Map improvement of *G. max* sequence by filling gaps

	FPC map	gmax1.01		MTP
No. of gaps	1893	377		835
No. of gaps filled out by	148	126	152	160
	(4x draft sequence)	(FPC clones including unpaired BES)	(all the fingerprinted clones including unpaired BES)	(clones with unpaired BES)

FPC, FingerPrinted Contigs; MTP, minimum tiling path; BES, BAC end sequences.

There are many gaps represented as a series of Ns in the 8x soybean sequence (gmax1.01). A total of 1000 Ns indicate gaps between scaffolds that were not spanned using the Arachne assembler, 100 Ns indicate gaps without length information, and a specific number of Ns indicate gaps of known size (Figure 2). We assumed that a BAC clone would span at least part of a gap when one BES aligned near the edge of a contig abutting the gap and the clone pointed into the sequence gap. Some of the larger gaps with thousands of Ns were spanned by BAC clones with paired and/or unpaired BES by blast searching against the physical map already integrated with the 8x draft sequence data. To increase the coverage of the MTP picked from the clones building the FPC map, the physical location of the gaps on the FPC map were checked and the clones with unpaired BESs corresponding to the location were added to the MTP.

## Results

### BAC libraries

Three *Glycine max* cv. Williams 82 BAC libraries, GM_WBa, GM_WBb, and GM_WBc (http://genome.arizona.edu), were made with three different restriction enzymes, *Hind*III, *Bst*yI, and *Eco*RI, respectively, to reduce the likelihood of missing parts of the genome attributable to cloning bias. All three libraries were used to construct the *G. max* physical map. A BAC library was constructed using *Hind*III for *Glycine soja* PI468916, called GSS_Ba. The average insert size of GM_WBa, GM_WBb, GM_WBc, and GSS_Ba were 150, 150, 131, and 150 kb and represent 5.4, 12, 10.9, and 12x coverage of each genome, respectively. Subsets of each library were fingerprinted for construction of the FPC maps (Table 1).

### FPC maps for *G. max* and *G. soja*

**Figure 1:**
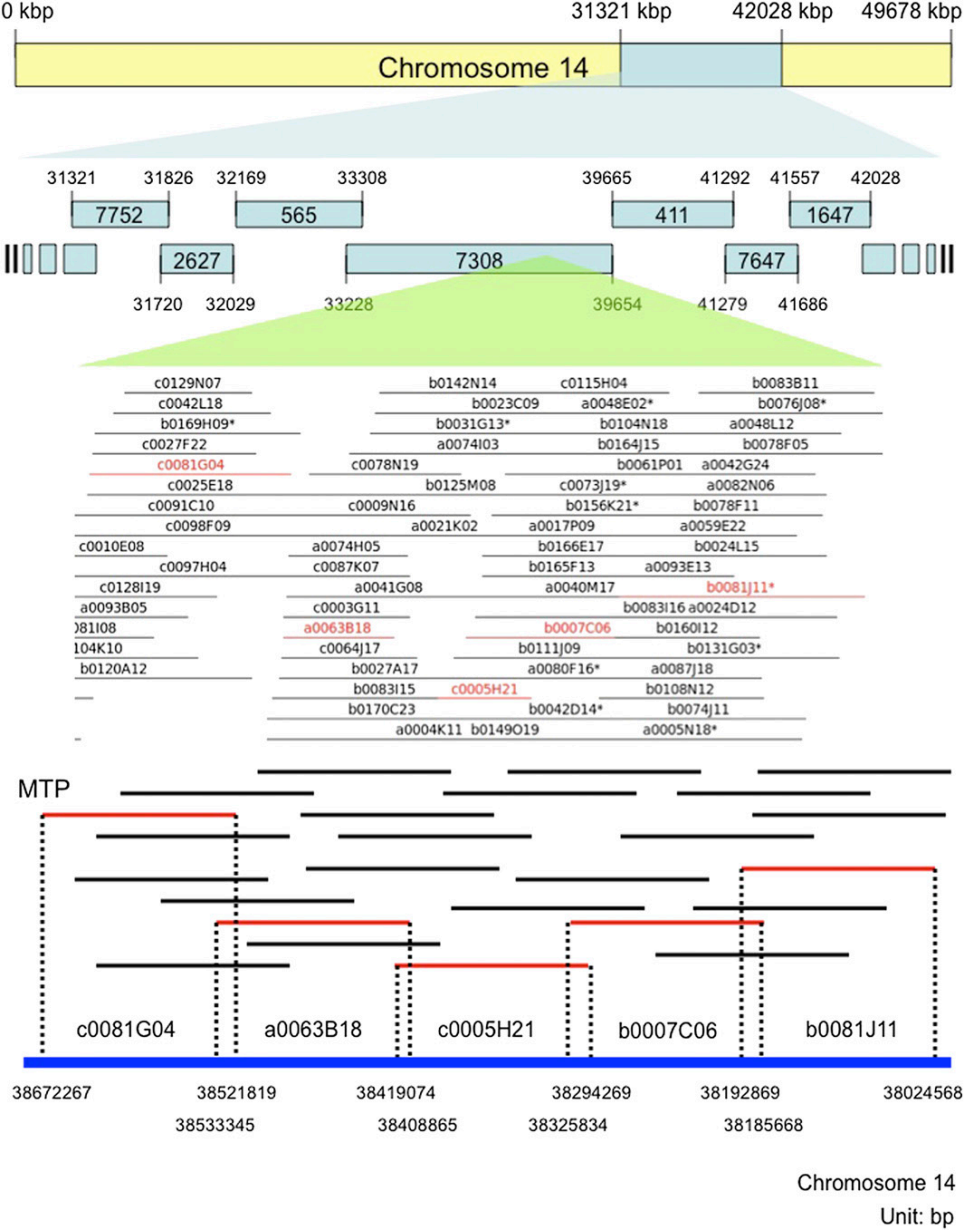
Schematic of picking a MTP from the *G. max* FPC map and chromosome-based pseudomolecules. BAC clones were aligned through the fingerprinting method, constructing contigs that were used to build chromosome-based pseudomolecules. These pseudomolecules were constructed based on MTP clones. The yellow bar represents chromosome 14, and blue fragments represent FPC contigs. The middle panel is a screenshot from the FPC program showing part of contig 7308. Each horizontal line represents a single BAC clone, and red lines represent clones used to construct the MTP. The bottom panel shows a schematic of FPC clones anchored to sequence map (blue line at bottom) with positions in base pairs. Red lines indicate clones chosen from the MTP.

Fingerprinted clones were clustered into contigs on the basis of their probability of coincidence score using the FPC software package (Soderlund *et al.* 1997, 2000). In total, 134,182 *G. max* and 81,247 *G. soja* BAC clones were used to construct the physical maps. A total of 107,214 *G. max* clones and 66,028 *G. soja* clones were ordered into contigs, and 26,968 and 15,219 clones remained as singletons (BACs that did not order into a contig), respectively (Table 4). Of the contigs, 1355 (78%) of *G. max*’s and 1053 (37%) of *G. soja*’s were ordered and oriented to 20 soybean chromosomes (Schmutz *et al.* 2010) using the alignment function of FPC (Nelson and Soderlund 2009). The aligned contigs spanned 838,932,828 bp for *G. max* and 547,374,187 bp for *G. soja* of the sequence length (87% of 967,233,029 bp and 58% of 950,068,807 bp, gmax 1.01; Figure 1). For the *G. max* alignment, unanchored sequence scaffolds were included in gmax 1.01, whereas for *G. soja*, only anchored scaffolds were used. In terms of the consensus FPC map, 607,788 and 426,033 cb units (Consensus Bands) were included in the aligned contigs for *G. max* and *G. soja*, respectively (93% of 648,007 cb units and 52% of 815,128; Table 5).

**Table 4:** Summary of clones and contigs used to construct the FPC maps

	*G. max*	*G. soja*
Valid fingerprints for FPC assembly	134,182	81,247
Total number of clones assembled	107,214	66,028
Contigs contain:		
>1000 clones	2	−
999-800 clones	5	3
799-600 clones	15	−
599-400 clones	29	2
399-200 clones	96	7
199-100 clones	105	52
99-50 clones	195	244
49-25 clones	271	511
24-10 clones	382	939
9-3 clones	350	892
2 clones	272	159
The number of singletons	26,968	15,219

FPC, FingerPrinted Contigs.

**Table 5:** Summary of FPC maps of *G. max* and *G. soja*

	*G. max*	*G. soja*
The number of contigs aligned	1355 (78% of 1722)	1053 (37% of 2809)
Total physical length of assembled contigs, bp	838,932,828 (87% of 967,233,029)	547,374,187 (58% of 950,068,807)
Total number of CB bands included in the contigs	607,788 (93% of 648,007)	426,033 (52% of 815,128)
Average number of bands per BAC	73.3	102.1
The number of markers anchored	4628	−

FPC, FingerPrinted Contigs; BAC, bacterial artificial chromosome.

### Genetic marker data for *G. max*

For a physical map to be useful in the assembly of a whole-genome sequence, it must be anchored to the genetic map (Jackson *et al.* 2006). A genetically anchored physical map is helpful not only for gene cloning but for a better understanding of genome structure that might confound a whole genome sequencing strategy (Shoemaker *et al.* 2008). Genetic markers and gene-based sequences from *G. max* were used to screen the BAC libraries (results available at http://www.soymap.org) to integrate the genetic and physical maps. The soybean genome sequence was then combined with the physical map using BES (Schmutz *et al.* 2010) so that the FPC contigs could be further integrated with the sequence and genetic maps. In this study, 4628 genetic markers consisting of 3952 SSR markers and 676 RFLP markers were anchored to the *G. max* physical map. Of these markers, 1725 were multiple-hit markers (MHM), indicating that the markers were anchored more than two BAC clones, 1181 MHM were linked to more than two contigs, 503 MHM were anchored to multiple clones on one contig, and 41 MHM were anchored to multiple singletons. The average number of contigs hit by the 3952 SSR markers was 1.5, and the average number of contigs hit by 676 RFLP markers was 1.6. Of 3952 SSR markers, 417 hit 0 contigs, 2601 hit 1 contig, 301 hit 2 contigs, and 633 markers hit more than 2 contigs. Of the 676 RFLP markers, 98 hit 0 contigs, 331 hit 1 contig, 145 hit 2 contigs, and 102 hit more than 2 contigs (Table 6). There were many MHM primarily as the result of the short sequences used to screen the BAC libraries and the duplicated soybean genome; however, these data are useful for confirmation of clone order and contig integrity and alignment to the sequence map.

**Table 6:** Summary of markers anchored to the FPC map of *G. max*

	No. Contigs					No. Clones						
	Avg.	0	1	2	>2	Avg.	1	<5		<10		≥10
SSR	1.5	417	2601	301	633	2.7	2698	451		631		172
RFLP	1.6	98	331	145	102	3.5	205	306		125		40
MHM		41	503	1181		Total	1725					

FPC, FingerPrinted Contigs; SSR, simple sequence repeat. RFLP, restriction fragment length polymorphism; MHM, multiple-hit markers.

### Minimum tiling path (MTP) for *G. max*

Four paradigms have been used to pick minimal tiling paths from FPC fingerprint maps. The first is a map-based approach. Fingerprints of clone pairs that appear to have minimal overlap are analyzed in the FPC gel image display (Coulson *et al.* 1986). The second is a BES-based approach in which a seed clone is selected and sequenced. This sequence is used to query a BES database to find a minimally overlapping clone; the process is then repeated iteratively (Venter *et al.* 1996). The third is a hybrid of the first two in which the seed clone selecting and extending process is the same as mentioned previously but the overlap is verified using a map-based approach to reduce the risk of false-positive overlaps (Marra *et al.* 1999). The fourth approach makes use of both BES and existing genomic sequence by using BES-to-sequence alignments to estimate BAC overlaps more accurately than is possible from fingerprint overlaps alone. Functions to implement this approach are built into FPC (Nelson and Soderlund 2009).

In the case of soybean, a genome sequence data (gmax 1.01) is already available. We integrated the sequence with the FPC map to build BAC-based pseudomolecules representing the 20 soybean chromosomes (http://soybase.org). Therefore, our MTP does not need to be “minimal” in the sense of budget constraints for BAC sequencing, and we instead selected BAC clones with the greatest reliability while attempting to minimize overlap between adjacent BACs (Figure 1). Two types of MTPs were picked from two different clone pools: (A) using only the clones contained in the FPC map; and (B) using all the clones from all three BAC libraries that had BESs, which may have been excluded from the FPC map because of fingerprinting errors (hereafter referred to as clone pools A and B, respectively). In the first approach, proximity in FPC provides an additional confirmation of overlapping MTP clones; however, a number of clones that have BES are not contained in the FPC map because of fingerprinting failures.

**Figure 2:**
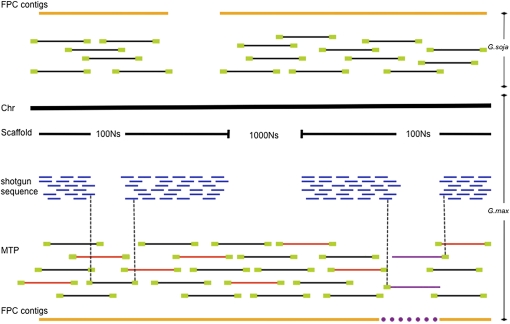
Representation of integration of the *G. max* draft sequence and the physical maps of *G. max* and *G. soja*. By integrating the draft sequence and the physical maps, gaps in the sequence could be spanned using clones from the physical maps based on BES and gaps in physical map can be spanned by the sequence map. By adding clones with unpaired BES, gaps existing in both the sequence and the physical maps were filled. The yellow bold lines indicate FPC contigs from both physical maps. The black bold line (Chr) represents a sequence scaffold from gmax1.01, and blue fragments represent shotgun sequences that are part of a sequence scaffold. Black and red lines represent BAC clones and green boxes represent BESs. Red lines indicate BAC clones from the MTP. Purple lines indicate the clones with unpaired BESs. Purple dotted line represents a gap that can be partially filled or spanned by adding clones with unpaired BESs.

FPC provides an approximation of where clones should be relative to one another in a contig as there may be error in the band calling of individual clones or in the determination of clone overlap. Therefore, for the clone-ordering process, clones may not end up in the FPC map although BESs can be used to order clones relative to the genome sequence. Thus, we used not only the FPC clones but also the clones not in FPC but having BESs to improve the accuracy of the BAC-based maps. The MTP with only FPC clones consists of 1422 GM_WBa, 3887 GM_WBb, and 2035 GM_WBc BAC clones containing 914 gaps and an average of 21.9 kbp overlap between clones. The MTP with all the fingerprinted clones, even those not in FPC contigs, comprises 1019 GM_WBa, 3095 GM_WBb, and 2969 GM_WBc clones with 835 gaps and an average of 22.1 kbp overlap between clones (Table 7). To attempt to span gaps in the sequence scaffolds, clones with unpaired BES were added to MTPs. BACs with unpaired BES were anchored to MTP only when they aligned near the edge of a contig pointing toward the gap (Figure 2). In the MTP composed of clones only in the FPC map, 146 gaps were spanned by clones with unpaired BESs and the average overlapping region was elongated by an average of 1.5 kbp. In the MTP built with all three BAC libraries, 160 gaps were covered by the clones with unpaired BESs and the BAC overlaps were extended by an average of 1.4 kbp (Tables 3 and 7).

**Table 7:** The number and characteristics of *G. max* BAC clones used for picking MTP

Library	No. Clones in MTP			
	FPC Clones/Paired BES	FPC Clones/Unpaired BES	All Clones/Paired BES	All Clones/Unpaired BES
GM_WBa	1422	1477	1019	1064
GM_WBb	3887	4034	3095	3218
GM_WBc	2035	2086	2969	3045
Total	7344	7597	7083	7327
Gaps	914	768	835	675
Avg. of overlap	21,942 bp	23,419 bp	22,094 bp	23,526 bp

BAC, bacterial artificial chromosome; MTP, minimum tiling path; BES, BAC end sequences; FPC, FingerPrinted Contigs;

### Alignment of *G. soja* BESs to *G. max* genome sequence

**Figure 3:**
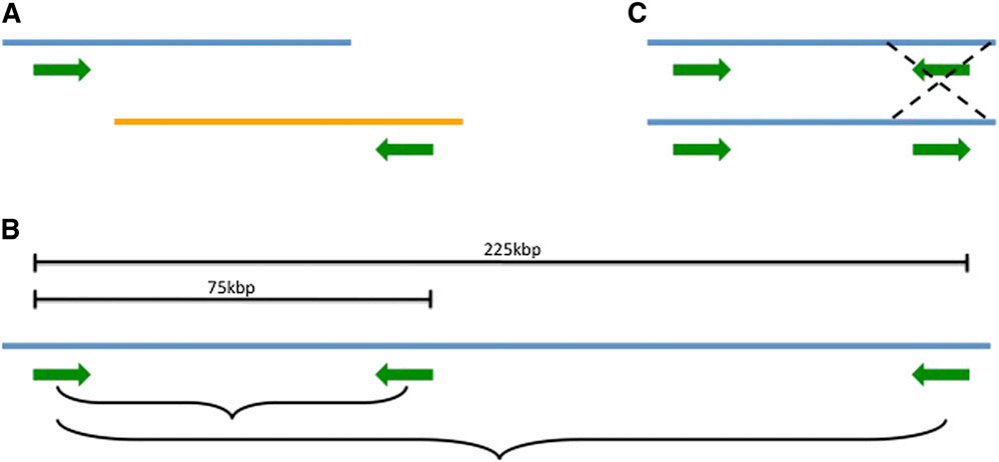
Schematic of detecting rearrangements using mapped BES. (A) Potential translocation where paired BESs map to different chromosomes (blue and yellow). (B) Size distribution to show insertions/deletions. Expected range is 75 kbp to 225 kbp. Mapped pairs of BESs outside this range are predicted to have either insertions or deletions. (C) Potential inversion where paired BESs shown as expected on top (inverted relative to each other) are pointing the same direction on bottom.

*G. soja*’s BES were aligned to *G. max*’s whole-genome sequence (gmax1.01) to detect structural difference between *G. max* and *G. soja*. Of 180,099 total BESs, 88,950 clones have paired end sequences, and 2199 clones have sequence for one end only (Table 8). Alignments of these BESs to the gmax1.01 genome resulted in 2675 of the 88,905 clones having only one end aligned to the reference genome. A majority of the clones, 67,047, had BESs that could be aligned to the same chromosome; however, 19,143 clones had BESs that aligned to different chromosomes, indicative of potential rearrangements (Figure 3A).

**Table 8:** Alignment of *G. soja* BESs against the *G. max* genome sequence

	No. Clones
Total Number of *G. soja* BES	180,099
Clones with unpaired BES	2199
Clones with paired BES	88,905
Clones where only one end aligned	2675
Clones where BES aligned to different chromosomes	19,143
Clones where BES aligned to same chromosome	67,047
75 kbp < clones < 225 kbp	59,899
Clones < 75 kbp	3352
Clones > 225 kbp	1965
Clones with BES with expected orientation	63,888
Clones with BES in opposite direction	1184
Clones with BES same direction	1975

BES, BAC end sequences.

By examining the distance and orientation of paired BESs, we were able to look at intrachromosomal rearrangements. BES pairs when aligned to the genome should be inverted relative to each other (sequencing from either end of the cloning vector) and we expected the distance between the ends to be within 75 to 225 kbp of each other (Figure 3B). Of the 67,047 clones where paired BESs aligned to same chromosomes, 89.3% (59,899) were within a range of 75 kbp to 225 kbp, 2.9% (1965) were greater than 225 kbp, and 5.0% (3352) less than 75 kbp apart (supporting information, Figure S1). BAC clones where paired BESs aligned more than 1.5 Mbp apart were excluded as potential artifacts. Of 3796 clones, 1965 were included within 225-kbp to 1.5-Mbp range. A majority of clones fell within the expected distance of an average BAC library insert distribution although there were many clones that had potential insertions/deletions.

In terms of orientation of BESs where both BESs were located on same chromosome, 63,888 clones had the expected orientation (BESs pointing toward each other; Figure 3C). A total of 1184 clones had BESs pointing in the opposite direction, and another 1975 clones had BESs pointing in the same direction, indicative of potential inversions (Table 8).

## Discussion

### The MTP with the fewest gaps and the most coverage Over *G. max* genome sequence

To increase the coverage of the physical map but maintain reliability, three approaches were considered. First, the preliminary FPC map was integrated with whole-genome draft sequence, meaning that the draft sequence was aligned to the FPC contigs via BES alignments. A number of FPC contigs were merged based with this approach, and 148 gaps in the FPC map were closed (Table 3). This was done using the preliminary 4x sequence assembly from the Joint Genome Institute–Stanford Human Genome Center, using the Arachne assembler (Batzoglou *et al.* 2002; Jaffe *et al.* 2003); later assemblies did not yield additional FPC merges.

Second, to increase coverage of the sequence map, clones with unpaired BES were added to the draft sequence and to the MTP (Figure 2). The 8x draft sequence (gmax1.01) that consists of 20 scaffolds covering 950,068,807 bp of sequence length has 377 gaps indicated with 1000 Ns that are not spanned by only paired BES information (Table 3). Nearly one-third, 126 of 377 sequence gaps (33.4%), were spanned by BAC clones from the clone pool A (only the clones contained in the FPC map) with or without paired BESs and an additional 26 gaps (152 of 377, 40.3%) by clones from clone pool B (all fingerprinted clones from the three BAC libraries) with or without paired BES. The MTP picked from all the fingerprinted clones (clone pool B) with paired BESs had 835 gaps of which 160 were covered by adding 244 clones with unpaired BES resulting in additional coverage of as much as 5,947,374 bp. In the case of the MTP picked from only the FPC clones (clone pool A), 6,457,374 bp was covered from clones with unpaired BESs.

Finally, four different MTPs were picked from two different BAC clone pools to maximize coverage and minimize gaps: (1) FPC clone pool A, in which all BACs have paired BESs; (2) FPC clone pool A, in which BACs have both paired and unpaired BESs; (3) Pool B of all three BAC libraries, in which BACs have paired BESs; and (4) Pool B of all three BAC library, in which BACs have both paired and unpaired BESs (Table 7). Comparing MTPs 3 and 4 to 1 and 2, ∼ 80 sequence gaps were spanned, and the average length of overlap was similar. Because only ∼60% of the three BAC libraries (134,182/223,640) were used to construct the FPC map, there were more options with the larger pools to select clones that had more sequence coverage and less overlap with adjacent clones. Thus, when all clones were used, the number of clones used to build the MTPs decreased and the coverage length increased. When only clones with paired BES were used, it increased by 14,561,053 bp (from MTP1 to MTP3), and when both paired and unpaired BES were utilized, it increased by 14,050,753 bp (from MTP2 to MTP4).

Comparing MTPs 2 and 4 to 1 and 3, in terms of BESs, ∼140 gaps were spanned, and the average length of overlap was increased by ∼1.4 kbp. Once an MTP was picked using clones with paired BES, clones with unpaired BES were used only where we were unable to place clones with paired BESs. Therefore, it was reasonable that both the total numbers of clones used to build the MTPs and the average lengths of overlap increased. The sum of gaps covered by the clones with unpaired BES in both pools was 306, which was ∼1.8 times more than the sum of gaps spanned when MTPs were picked in the larger pool with all the three BAC libraries.

We conclude that the MTP selected using all the three BAC libraries containing clones with paired and unpaired BES is the best in that it had fewer gaps and the greatest coverage of the sequence map. In instances in which users need to know the relative locations of clones, this can be inferred through the FPC map constructed using clones with both paired and unpaired BESs. This high-resolution chromosome-anchored physical map will serve as an important tool for (1) improving the genome sequence by spanning gaps (in progress); (2) resolving assembly errors caused by repetitive sequences, large gene families and segmental duplications; (3) map-based cloning; and (4) cloning sequences that are too large or repetitive for polymerase chain reaction−based cloning (http://soybase.org).

### The physical map of *G. soja* parallel to *G. max* genome sequence

FPC-based physical maps were originally made to assist in clone-by-clone sequencing by identifying minimal tiling paths; indeed, the maize FPC map was used for this purpose as recently as 2009 (Schnable *et al.* 2009). In the case of whole-genome shotgun sequencing, physical maps may be used for closing sequence gaps, confirmation of the sequence assembly, and to provide an anchored, clone-based resource for further research. With the transition to “next-generation” sequencing technologies, BAC-based maps can be even more crucial for ordering sequence contigs/scaffolds and confirming assemblies (Mardis 2008; Shendure and Ji 2008). Wild soybean, *G. soja*, genome was sequenced using the Illumina Genome Analyzer resulting in 48.8 Gbp of sequence, ∼52-fold sequence coverage of the genome. The short reads (35 or 76 bp) were mapped to gmax1.01 reference for assembly (Kim *et al.* 2010). Although it covered ∼43-fold of the reference genome, structural differences between two genomes were difficult to analyze because of the short read lengths and short distances between paired reads (Findley *et al.* 2010; Mahama *et al.* 1999; Yang *et al.* 2008).

Putative chromosomal structural rearrangements between *G. soja* and *G. max* could be detected through the alignment of BESs from *G. soja* against the *G. max* reference sequence (gmax 1.01; Table 8). BAC clones in which paired BESs aligned to different chromosomes indicate potential translocations; however, this interpretation is complicated by recent polyploidy events that occurred in the genus glycine. Insertions and deletions could be predicted from clones where paired BESs aligned too far (>225 kb) or too close (<75 kb) from each other on a chromosome. Inversions were predicted from paired BESs that pointed in either the opposition or same direction, as opposed to the expected orientation of toward each other (Figure 3). The average insert size of paired BESs between 75 kbp and 225 kbp was 146 kbp, consistent with the average insert size of GSS_Ba *G. soja* library (150 kbp; Table 1). The average insert size of paired BESs greater than 225 kbp was ∼445 kbp and less than 75 kbp was ∼37 kbp (Figure S1). This is an underestimate because small insertions or deletions would be missed because of the variability in BAC insert sizes. However, we were able to calculate a rough estimate of how much of the genome might be in flux between the two species (Kim *et al.* 2007). Considering insertions and deletion only, we estimate that at least 998 kbp is flux between *G. soja* and the domesticated *G. max*. The estimated sizes of insertions and deletions were ∼300 kbp and 110 kbp, respectively, and deletions were 71% more frequent than insertions. A few hotspots for insertions, deletions, and inversions were detected on the *G. max* chromosomes (Figure S2).

The importance of wild soybean (*G. soja*) as genetic resource for potentially valuable genes for introgression into soybean cannot be overstated. This was the reasoning for the sequencing of *G. soja* accession IT182932 as well another 17 other accessions of wild soybean (to 5x sequence coverage) (Kim *et al.* 2010; Lam *et al.* 2010). The sequence similarity between *G. max* and *G. soja* is ∼98%; however, structural differences are not captured in this statistic. Reciprocal translocations, segmental duplications, and insertions/deletions complicate the ability to map *G. soja* using *G. max* as a reference and short read WGS does not currently capture this information. Thus, physical maps remain useful for investigating and describing structural evolution that has occurred between these two genomes and to allow researchers to effectively shuttle between the genomes to capture useful genetic information for crop improvement and basic genetics.

## Supplementary Material

Supporting Information

## References

[bib1] Batzoglou S. Jaffe D. B. Stanley K. Butler J. Gnerre S., 2002 Arachne: a whole-genome shotgun assembler. Genome Res. 12: 177–189 11779843 10.1101/gr.208902 PMC155255

[bib2] Bouck J. B. Metzker M. L. Gibbs R. A., 2000 Shotgun sample sequence comparisons between mouse and human genomes. Nat. Genet. 25: 31–33 10802652 10.1038/75563

[bib3] Chen M. S. Presting G. Barbazuk W. B. Goicoechea J. L. Blackmon B., 2002 An integrated physical and genetic map of the rice genome. Plant Cell 14: 537–545 11910002 10.1105/tpc.010485 PMC150577

[bib4] Cone K. C. McMullen M. D. Bi I. V. Davis G. L. Yim Y. S., 2002 Genetic, physical, and informatics resources for maize. On the road to an integrated map. Plant Physiol. 130: 1598–1605 12481043 10.1104/pp.012245 PMC1540265

[bib5] Coulson A. Sulston J. Brenner S. Karn J., 1986 Toward a physical map of the genome of the nematode *Caenorhabditis-elegans*. Proc. Natl. Acad. Sci. U S A 83: 7821–7825 16593771 10.1073/pnas.83.20.7821 PMC386814

[bib6] Dijkstra E. W., 1959 A note on two problems in connection with graphs. Numer. Math. 1: 131–171

[bib7] Findley S. D. Cannon S. Varala K. Du J. C. Ma J. X., 2010 A fluorescence in situ hybridization system for karyotyping soybean. Genetics 185: 727–744 20421607 10.1534/genetics.109.113753 PMC2907198

[bib8] Gepts P. Beavis W. D. Brummer E. C. Shoemaker R. C. Stalker H. T., 2005 Legumes as a model plant family. Genomics for food and feed report of the cross-legume advances through genomics conference. Plant Physiol. 137: 1228–1235 15824285 10.1104/pp.105.060871 PMC1088316

[bib9] Hoskins R. A. Nelson C. R. Berman B. P. Laverty T. R. George R. A., 2000 A BAC-based physical map of the major autosomes of Drosophila melanogaster. Science 287: 2271–2274 10731150 10.1126/science.287.5461.2271

[bib10] Hyten D. L. Song Q. J. Zhu Y. L. Choi I. Y. Nelson R. L., 2006 Impacts of genetic bottlenecks on soybean genome diversity. Proc. Natl. Acad. Sci. U S A 103: 16666–16671 17068128 10.1073/pnas.0604379103 PMC1624862

[bib11] Jackson S. A. Rokhsar D. Stacey G. Shoemaker R. C. Schmutz J., 2006 Toward a reference sequencing of the soybean genome: a multiagency effort. Crop Sci. 46: S55–S61

[bib12] Jaffe D. B. Butler J. Gnerre S. Mauceli E. Lindblad-Toh K., 2003 Whole-genome sequence assembly for mammalian genomes: Arachne 2. Genome Res. 13: 91–96 12529310 10.1101/gr.828403 PMC430950

[bib13] Kim H. Miguel P. S. Nelson W. Collura K. Wissotski M., 2007 Comparative physical mapping between *Oryza sativa* (AA genome type) and *O. punctata* (BB genome type). Genetics 176: 379–390 17339227 10.1534/genetics.106.068783 PMC1893071

[bib14] Kim M. Y. Lee S. Van K. Kim T. H. Jeong S. C., 2010 Whole-genome sequencing and intensive analysis of the undomesticated soybean (Glycine soja Sieb. and Zucc.) genome. Proc. Natl. Acad. Sci. U S A 107: 22032–22037 21131573 10.1073/pnas.1009526107 PMC3009785

[bib15] Lam H. M. Xu X. Liu X. Chen W. B. Yang G. H., 2010 Resequencing of 31 wild and cultivated soybean genomes identifies patterns of genetic diversity and selection. Nat. Genet. 42: 1053–1059 21076406 10.1038/ng.715

[bib16] Lewin H. A. Larkin D. M. Pontius J. O’Brien S. J., 2009 Every genome sequence needs a good map. Genome Res. 19: 1925–1928 19596977 10.1101/gr.094557.109 PMC2775595

[bib17] Mahama A. A. Deaderick L. M. Sadanaga K. Newhouse K. E. Palmer R. G., 1999 Cytogenetic analysis of translocations in soybean. J. Hered. 90: 648–653

[bib18] Mardis E. R., 2008 Next-generation DNA sequencing methods. Annu. Rev. Genomics Hum. Genet. 9: 387–402 18576944 10.1146/annurev.genom.9.081307.164359

[bib19] Marra M. Kucaba T. Sekhon M. Hillier L. Martienssen R., 1999 zA map for sequence analysis of the *Arabidopsis thaliana* genome. Nat. Genet. 22: 265–270 10391214 10.1038/10327

[bib20] McPherson J. D. Marra M. Hillier L. Waterston R. H. Chinwalla A., 2001 A physical map of the human genome. Nature 409: 934–941 11237014 10.1038/35057157

[bib21] Mozo T. Dewar K. Dunn P. Ecker J. R. Fischer S., 1999 A complete BAC-based physical map of the *Arabidopsis thaliana* genome. Nat. Genet. 22: 271–275 10391215 10.1038/10334

[bib22] Nelson W. Soderlund C., 2009 Integrating sequence with FPC fingerprint maps. Nucleic Acids Res. 37: e36 19181701 10.1093/nar/gkp034 PMC2655663

[bib23] Pampanwar V. Engler F. Hatfield J. Blundy S. Gupta G., 2005 FPC web tools for rice, maize, and distribution. Plant Physiol. 138: 116–126 15888684 10.1104/pp.104.056291 PMC1104167

[bib24] Pennisi E., 2000 Genomics. Mouse sequencers take up the shotgun. Science 287: 1179–1181 10712139 10.1126/science.287.5456.1179

[bib26] Schmutz J. Cannon S. B. Schlueter J. Ma J. Mitros T., 2010 Genome sequence of the palaeopolyploid soybean. Nature 463: 178–183 20075913 10.1038/nature08670

[bib27] Schnable P. S. Ware D. Fulton R. S. Stein J. C. Wei F., 2009 The B73 maize genome: complexity, diversity, and dynamics. Science 326: 1112–1115 19965430 10.1126/science.1178534

[bib28] Shendure J. Ji H. L., 2008 Next-generation DNA sequencing. Nat. Biotechnol. 26: 1135–1145 18846087 10.1038/nbt1486

[bib29] Shoemaker R. C. Polzin K. Labate J. Specht J. Brummer E. C., 1996 Genome duplication in soybean (Glycine subgenus soja). Genetics 144: 329–338 8878696 10.1093/genetics/144.1.329 PMC1207505

[bib30] Shoemaker R. C. Grant D. Olson T. Warren W. C. Wing R., 2008 Microsatellite discovery from BAC end sequences and genetic mapping to anchor the soybean physical and genetic maps. Genome 51: 294–302 18356965 10.1139/G08-010

[bib31] Soderlund C. Longden I. Mott R., 1997 FPC: a system for building contigs from restriction fingerprinted clones. CABIOS 13: 523–535 9367125 10.1093/bioinformatics/13.5.523

[bib32] Soderlund C. Humphray S. Dunham A. French L., 2000 Contigs built with fingerprints, markers, and FPCV4.7. Genome Res. 10: 1772–1787 11076862 10.1101/gr.gr-1375r PMC310962

[bib33] Stacey G. Vodkin L. Parrott W. A. Shoemaker R. C., 2004 National Science Foundation−sponsored workshop report. Draft plan for soybean genomics. Plant Physiol. 135: 59–70 15141067 10.1104/pp.103.037903 PMC429333

[bib35] Venter J. C. Smith H. O. Hood L., 1996 A new strategy for genome sequencing. Nature 381: 364–366 8632789 10.1038/381364a0

[bib36] Warren W. C., The Soybean Mapping Consortium, 2006 A physical map of the “William 82” soybean (Glycine max) genome, Volume Plant and Animal Genomes XIV Conference, San Diego, CA

[bib37] Wei F. Coe E. Nelson W. Bharti A. K. Engler F., 2007 Physical and genetic structure of the maize genome reflects its complex evolutionary history. PLoS Genet. 3: e123 17658954 10.1371/journal.pgen.0030123 PMC1934398

[bib38] Wei F. Zhang J. Zhou S. He R. Schaeffer M., 2009 The physical and genetic framework of the maize B73 genome. PLoS Genet. 5: e1000715 19936061 10.1371/journal.pgen.1000715 PMC2774505

[bib39] Wu C. Sun S. Nimmakayala P. Santos F. A. Meksem K., 2004 a A BAC- and BIBAC-based physical map of the soybean genome. Genome Res. 14: 319–326 14718376 10.1101/gr.1405004 PMC327108

[bib40] Wu C. C. Nimmakayala P. Santos F. A. Springman R. Scheuring C., 2004 b Construction and characterization of a soybean bacterial artificial chromosome library and use of multiple complementary libraries for genome physical mapping. Theor. Appl. Genet. 109: 1041–1050 15164176 10.1007/s00122-004-1712-y

[bib41] Yang K. Moon J. K. Jeong N. Back K. Kim H. M., 2008 Genome structure in soybean revealed by a genomewide genetic map constructed from a single population. Genomics 92: 52–59 18486440 10.1016/j.ygeno.2008.03.008

