# Supplementary material for: Integration of the Draft Sequence and Physical Map as a Framework for Genomic Research in Soybean (Glycine max (L.) Merr.) and Wild Soybean (Glycine soja Sieb. and Zucc.)
Source: G3 (Bethesda). 2012 Mar 1;2(3):321–9. doi: 10.1534/g3.111.001834 (PMC3291501; doi:10.1534/g3.111.001834)
Supplement: Supporting Information [file supp_2_3_321__index.html]

Supporting Information 

# Integration of the Draft Sequence and Physical Map as a Framework for Genomic Research in Soybean (*Glycine max* (L.) Merr.) and Wild Soybean (*Glycine soja* Sieb. and Zucc.)

## Supporting Information for Ha *et al*, 2012

**Files in this Data Supplement:**

- Supporting Information - Figures S1 and S2 (PDF, 1.1 MB)
- Figure S1 - *G. soja* BAC length distribution as aligned to the *G. max* sequence (gmax1.01) (PDF, 723 KB)
- Figure S2 - Heatmap showing putative structural variations between *G. max* and *G. soja* (PDF, 365 KB)
