# Supplementary material for: Integration of the Draft Sequence and Physical Map as a Framework for Genomic Research in Soybean (Glycine max (L.) Merr.) and Wild Soybean (Glycine soja Sieb. and Zucc.)
Source: G3 (Bethesda). 2012 Mar 1;2(3):321–9. doi: 10.1534/g3.111.001834 (PMC3291501; doi:10.1534/g3.111.001834)
Supplement: Supporting Information [file supp_2.3.321_001834SI.pdf]

(A)

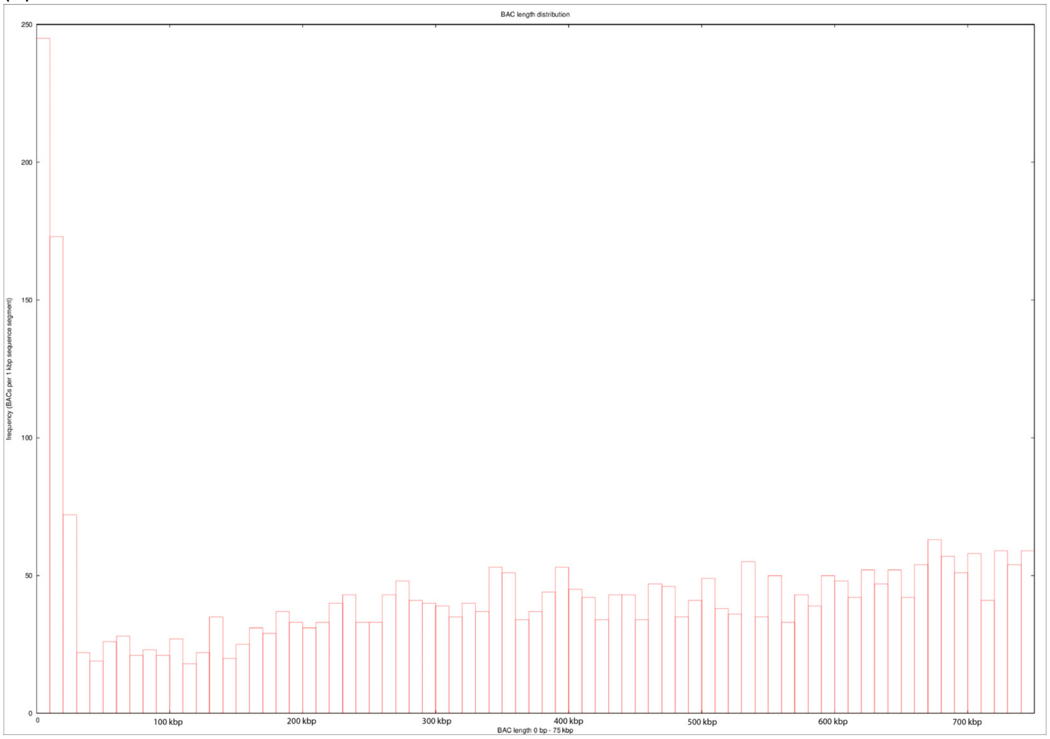
BAC length distribution
frequency (BACs per 1 kbp sequence segment)
250
200
150
100
50
0
0
100 kbp
200 kbp
300 kbp
400 kbp
500 kbp
600 kbp
700 kbp
BAC length 0 bp - 75 kbp

(B)

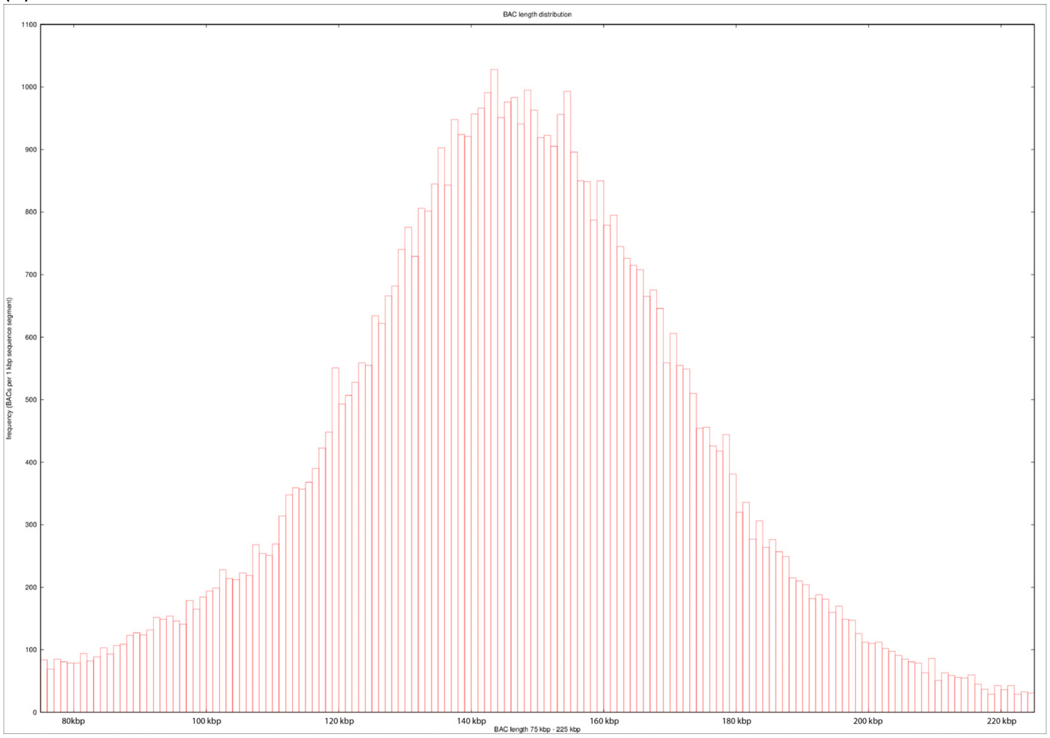
BAC length distribution
frequency (BACs per 1 kbp sequence segment)
1100
1000
900
800
700
600
500
400
300
200
100
0
80kbp
100 kbp
120 kbp
140 kbp
160 kbp
180 kbp
200 kbp
220 kbp
BAC length 75 kbp - 225 kbp

(C)

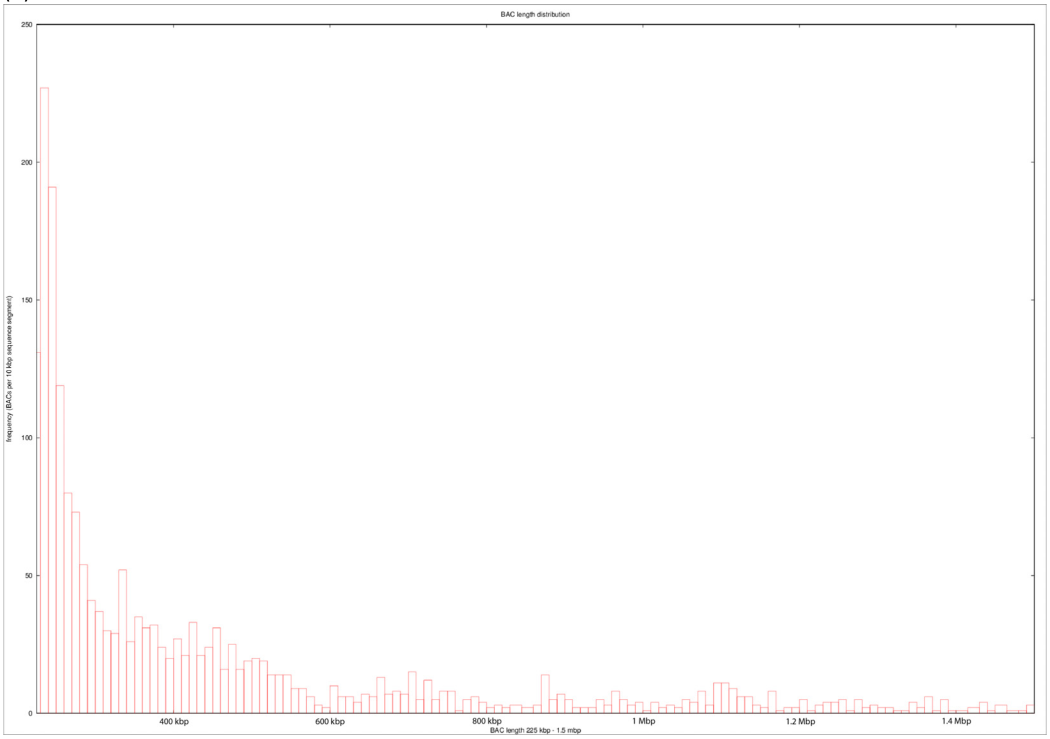

Figure S1 *G. soja* BAC length distribution as aligned to the *G. max* sequence (gmax1.01). Note that each graph uses different scales for X and Y axes. (A) The distribution of BAC clones where the distance between paired BESs were within 0-75 kbp of each other. Each bar on (A) and (B) indicates the number of BAC clones within a 1 kbp block. (B) The distribution of BAC clones where the distance between paired BESs were within 75-225 kbp of each other. (C) The distribution of BAC clones where the distance between paired BESs were within 225 kbp - 1.5 Mbp of each other. Each bar indicates the number of BAC clones within 10 kbp block.

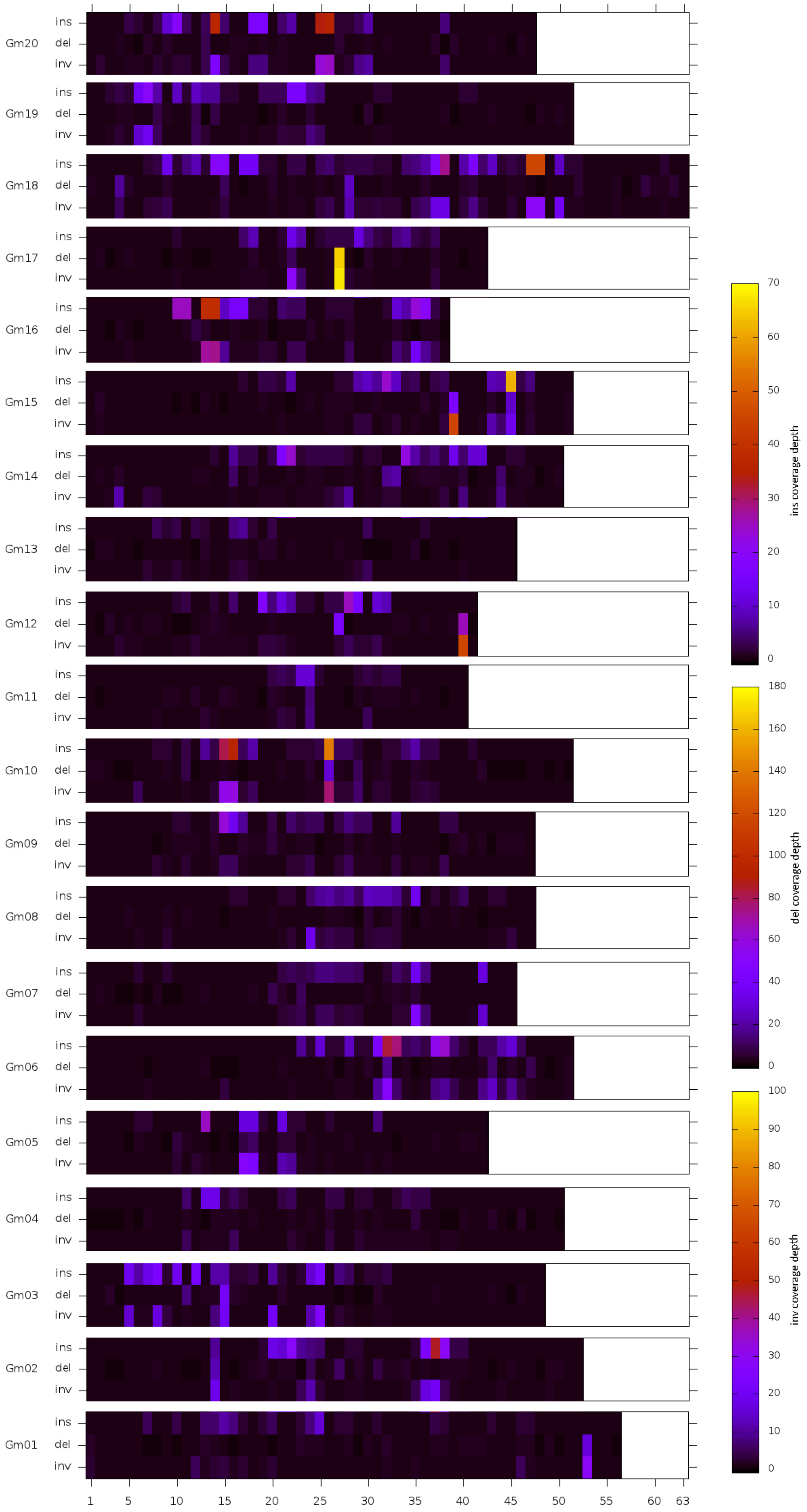
Gm20
Gm19
Gm18
Gm17
Gm16
Gm15
Gm14
Gm13
Gm12
Gm11
Gm10
Gm09
Gm08
Gm07
Gm06
Gm05
Gm04
Gm03
Gm02
Gm01
ins
del
inv
1 5 10 15 20 25 30 35 40 45 50 55 60 63
ins coverage depth
0 10 20 30 40 50 60 70
del coverage depth
0 20 40 60 80 100 120 140 160 180
inv coverage depth
0 10 20 30 40 50 60 70 80 90 100

Figure S2 Heatmap showing putative structural variations between *G. max* and *G. soja*. X axis indicates the physical location on the chromosomes in Mbp and y axis indicates 1 to 20 chromosomes of *G. max*. On Y axis, ins = insertions, del = deletions and inv = inversions. Note that ins, del and inv use different scale bars, on right.
