# Supplementary material for: Integration of the Draft Sequence and Physical Map as a Framework for Genomic Research in Soybean (Glycine max (L.) Merr.) and Wild Soybean (Glycine soja Sieb. and Zucc.)
Source: G3 (Bethesda). 2012 Mar 1;2(3):321–9. doi: 10.1534/g3.111.001834 (PMC3291501; doi:10.1534/g3.111.001834)
Supplement: Supporting Information [file supp_2.3.321_FigureS1.pdf]

(A)

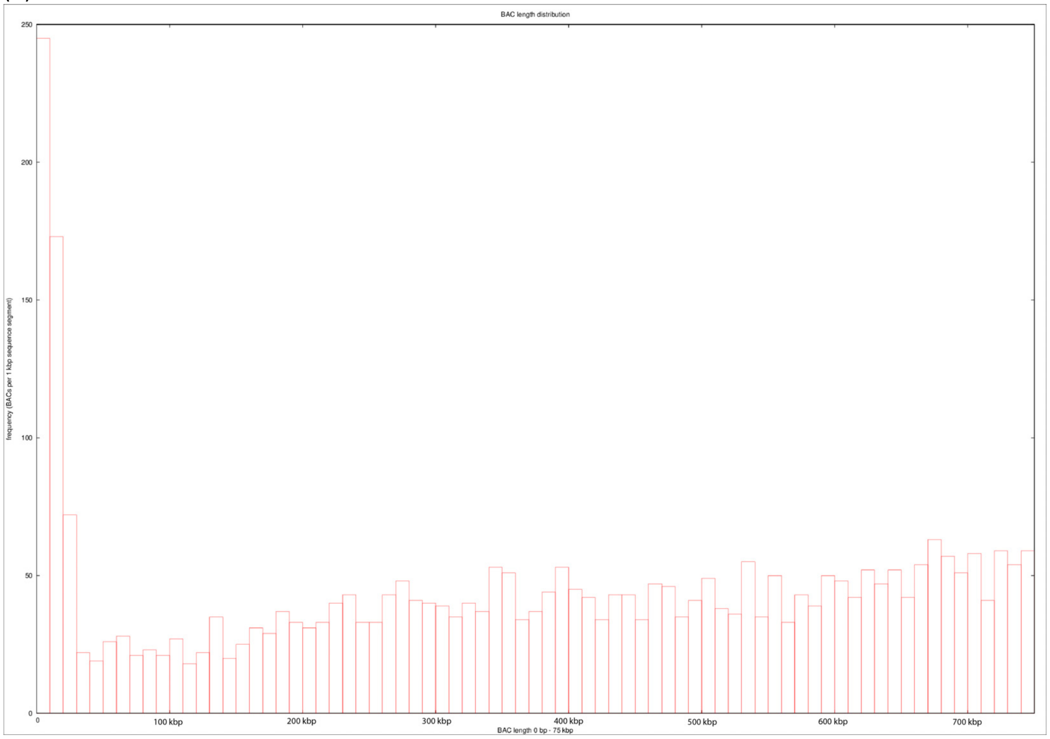
BAC length distribution
frequency (BACs per 1 kbp sequence segment)
BAC length 0 bp - 75 kbp
100 kbp
200 kbp
300 kbp
400 kbp
500 kbp
600 kbp
700 kbp

(B)

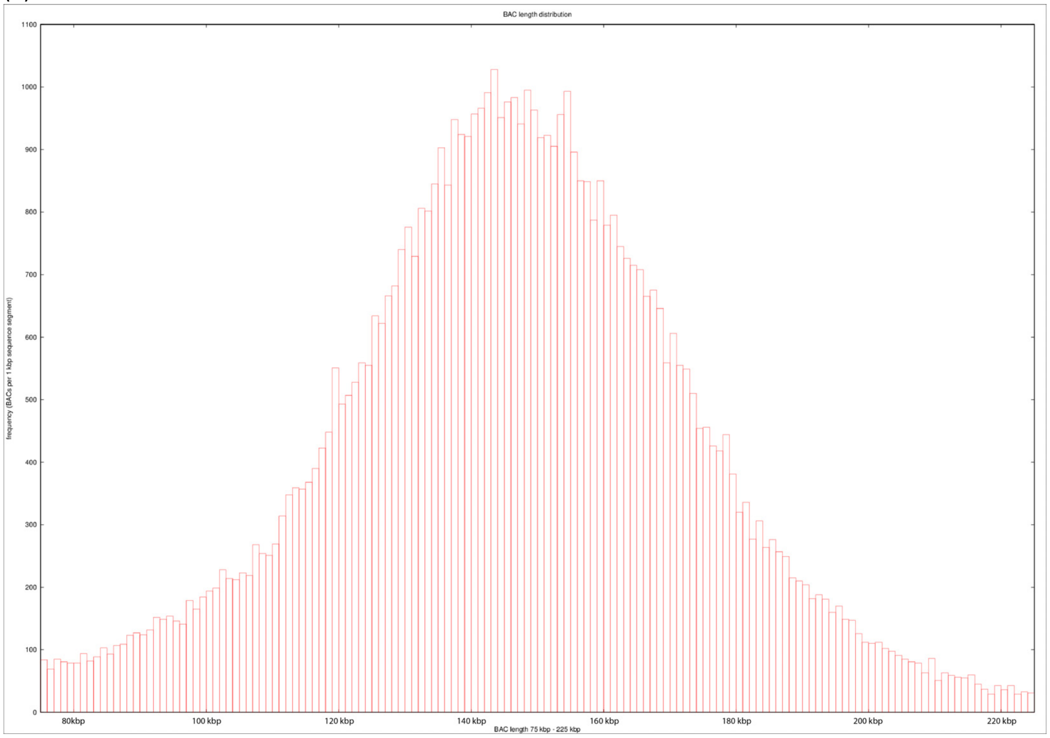
BAC length distribution
frequency (BACs per 1 kbp sequence segment)
BAC length 75 kbp - 225 kbp
80kbp
100 kbp
120 kbp
140 kbp
160 kbp
180 kbp
200 kbp
220 kbp

(C)

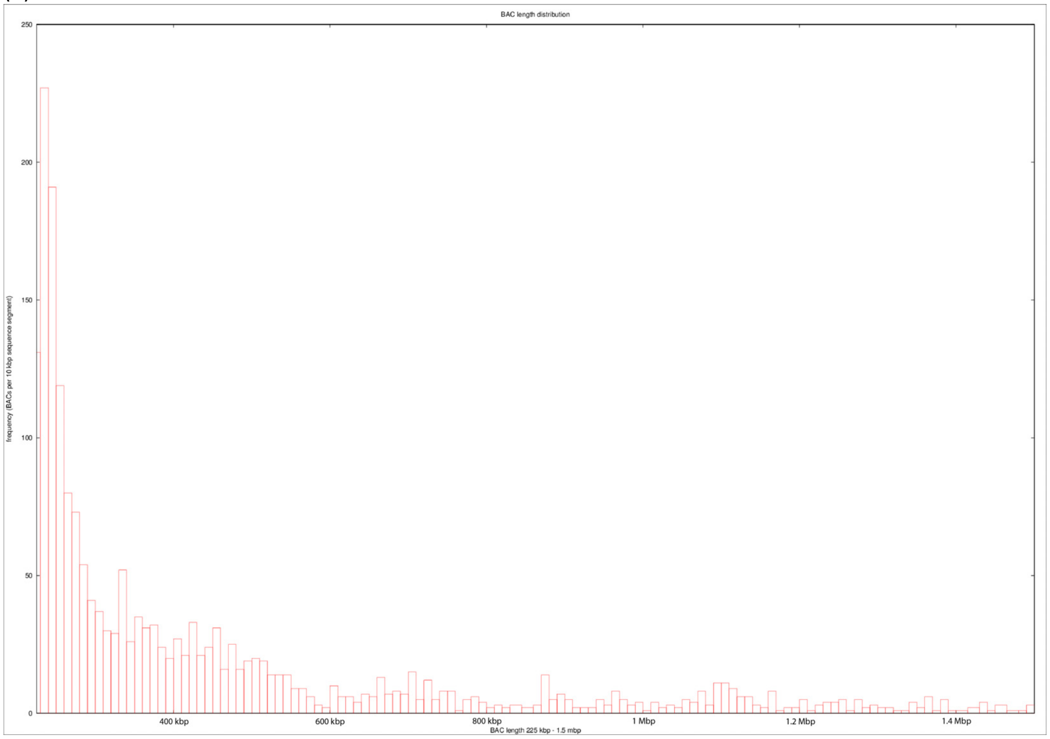

Figure S1 *G. soja* BAC length distribution as aligned to the *G. max* sequence (gmax1.01). Note that each graph uses different scales for X and Y axes. (A) The distribution of BAC clones where the distance between paired BESs were within 0-75 kbp of each other. Each bar on (A) and (B) indicates the number of BAC clones within a 1 kbp block. (B) The distribution of BAC clones where the distance between paired BESs were within 75-225 kbp of each other. (C) The distribution of BAC clones where the distance between paired BESs were within 225 kbp - 1.5 Mbp of each other. Each bar indicates the number of BAC clones within 10 kbp block.
