# Supplementary material for: Integration of the Draft Sequence and Physical Map as a Framework for Genomic Research in Soybean (Glycine max (L.) Merr.) and Wild Soybean (Glycine soja Sieb. and Zucc.)
Source: G3 (Bethesda). 2012 Mar 1;2(3):321–9. doi: 10.1534/g3.111.001834 (PMC3291501; doi:10.1534/g3.111.001834)
Supplement: Supporting Information [file supp_2.3.321_FigureS2.pdf]

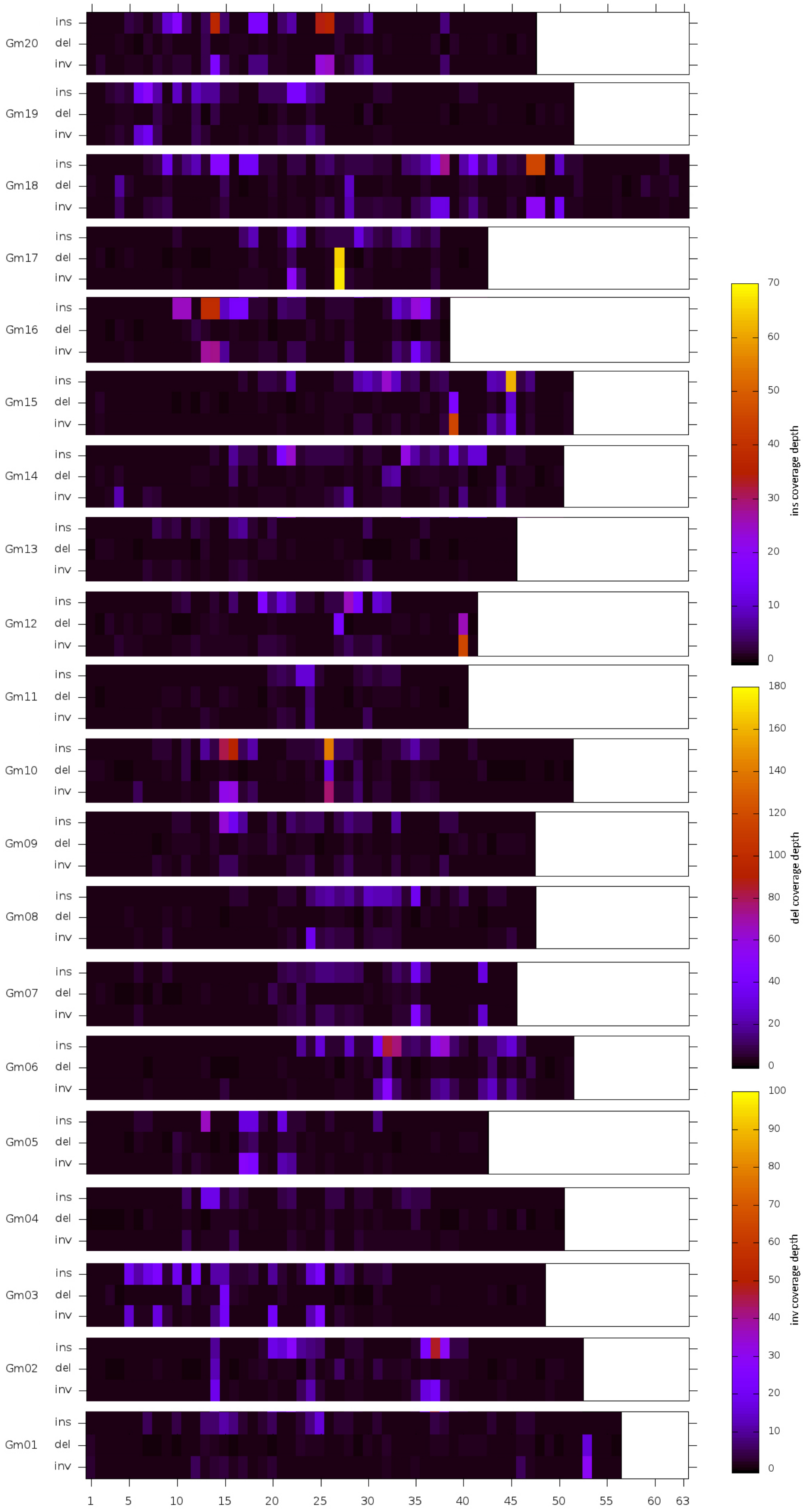
Gm20
Gm19
Gm18
Gm17
Gm16
Gm15
Gm14
Gm13
Gm12
Gm11
Gm10
Gm09
Gm08
Gm07
Gm06
Gm05
Gm04
Gm03
Gm02
Gm01
ins
del
inv
1 5 10 15 20 25 30 35 40 45 50 55 60 63
ins coverage depth
0 10 20 30 40 50 60 70
del coverage depth
0 20 40 60 80 100 120 140 160 180
inv coverage depth
0 10 20 30 40 50 60 70 80 90 100

**Figure S2** Heatmap showing putative structural variations between *G. max* and *G. soja*. X axis indicates the physical location on the chromosomes in Mbp and y axis indicates 1 to 20 chromosomes of *G. max*. On Y axis, ins = insertions, del = deletions and inv = inversions. Note that ins, del and inv use different scale bars, on right.
